# Quality of life of patients irradiated for head and neck cancer and impact of rehabilitation with a removable dental prosthetic: 1-year follow-up study

**DOI:** 10.4317/jced.59315

**Published:** 2022-03-01

**Authors:** Frédéric Silvestri, Bérengère Saliba-Serre, Michel Ruquet, Nicolas Graillon, Nicolas Fakhry, Abbas Mourad, Gérald Maille

**Affiliations:** 1 Ecole de Médecine dentaire, Aix-Marseille Université, 27 boulevard Jean Moulin, 13005 Marseille; Pôle Odontologie, UF Prothèses, Hôpital Timone, Assistance Publique des Hôpitaux de Marseille, 264 rue Saint-Pierre, 13385 Marseille Cedex 5, France. EFS CNRS, Faculté des Sciences Médicales et Paramédicales, UMR 7268 ADES, Aix-Marseille Université, 51 boulevard Pierre Dramard, 13944 Marseille Cedex 15, France; 2 EFS CNRS, Faculté des Sciences Médicales et Paramédicales, UMR 7268 ADES, Aix-Marseille Université, 51 boulevard Pierre Dramard, 13944 Marseille Cedex 15, France; 3 Faculté des Sciences Médicales et Paramédicales, Aix-Marseille Université, 27 boulevard Jean Moulin, 13005 Marseille; Pôle PROMO, Service de Chirurgie Maxillofaciale Stomatologie et Chirurgie Orale, Hôpital de la Conception, Assistance Publique des Hôpitaux de Marseille, 147 boulevard Baille, 13005 Marseille, France; 4 Faculté des Sciences Médicales et Paramédicales, Aix-Marseille Université, 27 boulevard Jean Moulin, 13005 Marseille; Pôle PROMO, Service ORL et Chirurgie Cervico-faciale, Hôpital de la Conception, Assistance Publique des Hôpitaux de Marseille, 147 boulevard Baille, 13005 Marseille, France; 5 UMR 912 SESSTIM, INSERM, Aix-Marseille Université, SanteRcom, Faculté des sciences médicales et paramédicales, 27 boulevard Jean Moulin, 13005 Marseille, France; 6 Ecole de Médecine dentaire, Aix-Marseille Université, 27 boulevard Jean Moulin, 13555 Marseille Cedex 5; Pôle Odontologie, UF Prothèses, Hôpital Timone, Assistance Publique des Hôpitaux de Marseille, 264 rue Saint-Pierre, 13385 Marseille Cedex 5, France. EFS CNRS, Faculté des Sciences Médicales et Paramédicales, UMR 7268 ADES, Aix-Marseille Université, 51 boulevard Pierre Dramard, 13944 Marseille Cedex 15, France

## Abstract

**Background:**

Head and neck cancer and its treatment cause significant functional, aesthetic, and social disabilities. These disabilities have a major impact on the quality of life of patients. When irradiation is required, removable dental prostheses are often the treatment of choice. This study investigated whether removable prosthetic rehabilitation improved patient function and aesthetics over the long term.

**Material and Methods:**

In this prospective study, we assessed quality of life in 78 patients with the General Oral Health Assessment Index (GOHAI) questionnaire. Assessments were performed before, and 1 week, 3 months, 6 months, and 12 months after denture insertion. We evaluated whether quality of life was influenced by the type of removable prosthesis and the primary tumour location.

**Results:**

We constructed mixed-effects linear regression models to identify correlates of the overall GOHAI score (GOAHI-add score) and the three domain-scores (functional, psychosocial, and discomfort/pain) in a longitudinal analysis over a 12-month follow-up. We compared scores (GOHAI-add score and domain-scores) in multivariate analyses between baseline (T0) and four post-insertion timepoints to determine significant changes.

**Conclusions:**

We found that removable prosthetic rehabilitation had an influence on the evolution of quality of life. The psychosocial component scores increased steadily over the year and changed more significantly than the functional and discomfort-pain components. The mandibular location of the primary lesion had a negative influence on quality of life. The type of removable prosthesis did not influence the results.

** Key words:**Quality of life, head and neck cancer, GOHAI, dental prosthesis, radiotherapy.

## Introduction

In 2017, in metropolitan France, the estimated numbers of new head and neck cancer (HNC) cases and HNC-related deaths were, respectively, 15,280 and 3630 (1). HNC and its treatment have a major functional impact on this anatomical region (i.e., impacts on chewing, swallowing, breathing, phonation, etc.). Surgical consequences (disfigurement) and adjuvant treatments (radiotherapy, chemotherapy) increase the functional consequences and generate psychological and social consequences (2) (3-5). Therefore, HNC has a substantial impact on quality of life, compared to other cancers (6,7).

There are several aspects related to quality of life, particularly oral quality of life. Oral quality of life depends on oral health, which was defined, in 2003, by the World Health Organization (8) as the “absence of oral or facial pain, oral or pharyngeal cancer, oral infection or injury, periodontal disease (disease affecting the gums), loosening and loss of teeth, and other diseases and disorders that limit a person’s ability to bite, chew, smile, and speak, and thus, their psychosocial well-being.” 

An essential component of managing patients with HNC is prosthetic rehabilitation. A prosthetic provides full or partial restoration of function and aesthetics (9). This restoration limits the loss of self-esteem and increases the capacity for physical and moral recovery (3). For patients that require irradiation for HNC, in most cases, a removable prosthesis is the treatment of choice (10).

Currently, we lack substantial evidence on the influence of a prosthesis on quality of life in patients with HNC (2,11). One study showed that a prosthesis improved the overall quality of life. That study followed patients for one year, but did not specify the extent of variation observed in the different quality of life domains, particularly the psychological component (12). A preliminary study showed that patient oral quality of life improved at 3 months after a prosthetic rehabilitation. However, that study did not distinguish among different types of prostheses or different tumour locations. Nevertheless, they observed a significant improvement in the psychosocial domain of quality of life (13).

The objective of this study was to investigate durable changes in the oral quality of life of patients for 1 year after a prosthesis was inserted. We evaluated whether clinical characteristics, the type of prosthetic rehabilitation, and the type of pathology led to changes in the oral quality of life and to what extents the different quality of life components were affected.

## Material and Methods

-Sample

We recruited 78 patients from the Functional Unit of the Hospital of Odontology (Pôle Odontologie) at the Hôpital Universitaire Timone in Marseille, France. Patients that had completed a therapeutic protocol for HNC were referred by the maxillofacial surgery and otolaryngology departments of different hospitals of the Assistance Publique des Hôpitaux de Marseille, for management, diagnosis, and oral rehabilitation.

Inclusion criteria were: a diagnosis of HNC, with no time limit; required radiotherapy as part of the treatment protocol (≥60 Gy), with no time limit; and a diagnosis that required oral rehabilitation with a removable prosthesis.

Exclusion criteria were: inability to complete the questionnaires; failure to wear the prosthesis.

-Ethical considerations 

Patients participating in this study were included in a clinical research protocol on oral health outcomes after upper aerodigestive tract radiotherapy (N IDCRB 2014-A01244-43). Administrative and medical data were collected directly from individuals. Informed consent forms were completed and signed by each participant. The study was conducted in compliance with the Declaration of Helsinki (14).

-Location of the primary tumour

The location of the primary tumour corresponded to the type of disability generated by the therapeutic protocol and the anatomic consequences related to the therapeutic protocol. Therefore, the pathologies were classified into three groups, based on the International Classification of Diseases 11th Revision (ICD-11) (15): Group A: maxillary tumours; Group B: mandibular tumours; and Group C: tumours in other locations (e.g., pharynx, larynx).

-Type of prosthesis

Patients were rehabilitated with removable dental prostheses. The heterogeneity among dental formulas led to the creation of groups of prostheses with common characteristics. Therefore, we assigned patients to one of two groups: patients with a rehabilitation diagnosis that required at least one removable complete prosthesis (full denture); and patients with a rehabilitation diagnosis that required a removable partial prosthesis (partial denture).

-Design

The oral quality of life was assessed at different time points with the General Oral Health Assessment Index (GOHAI) (16) (17). The GOHAI included 12 simple, easily understandable questions about the HNC context and the patient’s current state of vulnerability. The questions were scored on a scale of 1 to 5, and the overall score (GOHAI-add) ranged from 12 to 60 points. Scores ≤50 indicated a “poor” quality of life; scores between 51 and 56 indicated an “average” oral quality of life; and scores ≥57 indicated a “good” quality of life.

The GOHAI included the assessment of three domains. The functional domain (e.g., chewing, swallowing, speaking), which included 4 items, with scores from 4 to 20; the psychosocial domain (e.g., concern about one’s oral health, aesthetic dissatisfaction, and abandonment of social relationships), which included 5 items, with scores from 5 to 25; and the pain and discomfort domain, which included 3 items, with scores from 3 to 15.

The questionnaire was administered at five time-points: before the prosthesis was inserted (T0), and 1 week (T1), 3 months (T2), 6 months (T3), and 12 months (T4) after the prosthesis was inserted. A single practitioner administered the questionnaires, collected data, and performed the prosthetic rehabilitation, following the same protocols and techniques for each patient. The sample size was reduced over time, partly due to individuals lost to follow-up (i.e., death, absence from follow-up appointments, etc.) and partly due to insufficient time for data collection and management.

-Statistical analysis

For the descriptive analysis, patient characteristics are presented as numbers and percentages, for categorical data, or the median and interquartile range, for quantitative data. Wilcoxon rank sum tests were conducted to examine differences in GOHAI scores between patients that only completed the GOHAI at T0 and patients selected for the longitudinal study. The longitudinal study included GOHAI data from the T0-T4 follow-up period. We constructed mixed-effects linear regression models to identify correlates of the GOHAI add score and the three domain scores (functional, psychosocial, and discomfort/pain). Variables tested in the models included fixed variables, assessed at T0 (i.e., sex, age, type of pathology, and presence of at least one complete prothesis), and time-varying variables (i.e., items that changed between time-points). A likelihood ratio test, backward selection procedure was used to build the final multivariable models, which comprised only variables significant at the p≤0.05 level.

In these models, time was used as categorical variable. To identify factors that influenced the GOHAI-add and each domain-score, univariate and multivariate analyses were performed, and 95% confidence intervals (95%CIs) were calculated with the bootstrap resampling procedure.

Statistical analyses were performed with SAS 9.4 software (SAS Institute Inc. Cary. NC. USA) and R Software, version 3.6.3 (R Foundation for Statistical Computing. Vienna. Austria). All statistical tests were two-sided and the significance level was set to p≤0.05.

## Results

-Descriptive statistics

Initially, the study cohort included 78 patients; of these, 25 completed only the baseline assessment. Therefore, 53 patients were included in the longitudinal study. The initial and longitudinal study groups were not significantly different in socio-demographic or clinical characteristics. Moreover, the baseline GOHAI-add scores and the three domain-scores were not significantly different between the two groups.

•Characterization of the sample at T0

The longitudinal study consisted of 53 patients, aged 34 to 87 years, and 60.4% were women. The patient characteristics are summarized in Table 1. At T0, more than 90% of the patients stated that they had a poor oral quality of life, and only 2% stated that they had a good oral quality of life. The mean GOHAI-add score at T0 (data not shown) was 32.6 (sd=9.8), which indicated that the perceived oral quality of life was poor before prosthetic rehabilitation. At baseline, 39.6% of tumours were classified as group A and nearly half were classified as group B. At baseline, the sample was equally distributed between the two types of prosthesis.

**Table 1:**
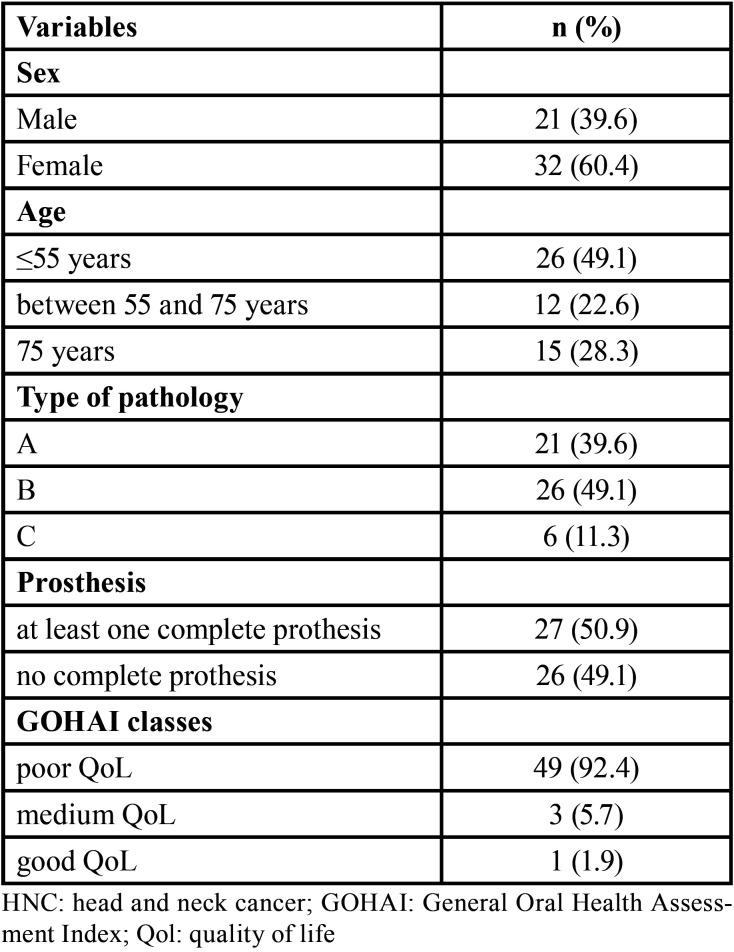
Baseline characteristics of patients treated for HNC that received prosthetic rehabilitation (n=53).

•Changes in the overall GOHAI score and the three GOHAI domain-scores over time

**Figure 1:**
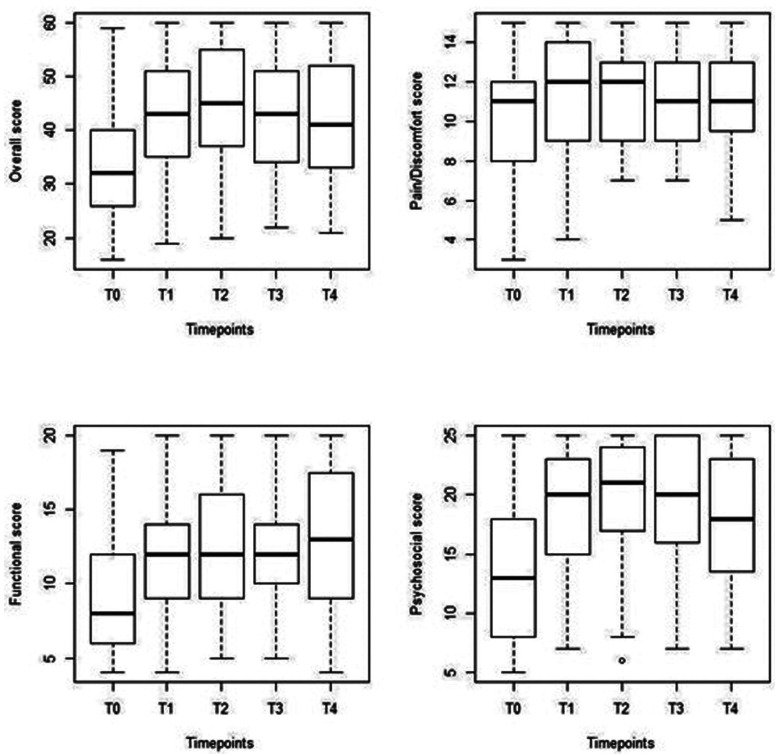
Boxplots show the overall GOHAI scores and the three domain-scores, measured at different timepoints. Overall GOHAI; Pain/Discomfort score; Functional score; Psychosocial score; GOHAI: General Oral Health Assessment Index; T0: before the day of prosthesis insertion; T1-T4: timepoints after prosthesis insertion, as follows: T1: 1 week, T2: 3 months, T3: 6 months, T4: 12 months.

For the GOHAI-add score and the three domain-scores, we observed that the median score at T1 was higher than that at T0 (Table 2, Fig. 1). However, from T1 to T3, there were very small variations in the quality-of-life scores.

**Table 2:**
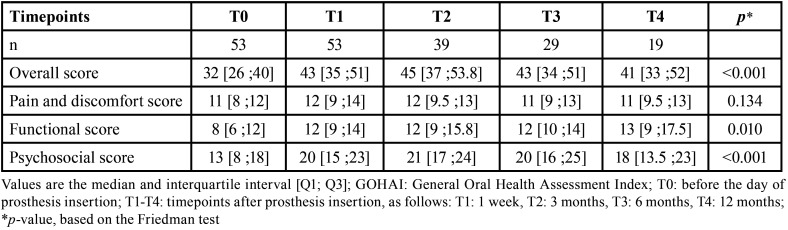
Overall GOHAI scores and domain-scores, measured at different time-points.

-Mixed-effect models

The mixed-effect model results are presented in  Table 3 and  Table 4. The univariate analysis based on the mixed-effect model showed that patient sex, age, and prothesis type had no significant effects on the overall GOHAI score (Table 3), the pain/discomfort score, or the psychological score (Table 4). On the other hand, the type of pathology significantly affected the overall GOHAI score and the pain and discomfort score. In contrast, the type of pathology did not significantly affect the functional score or the psychological score (Table 4).

**Table 3:**
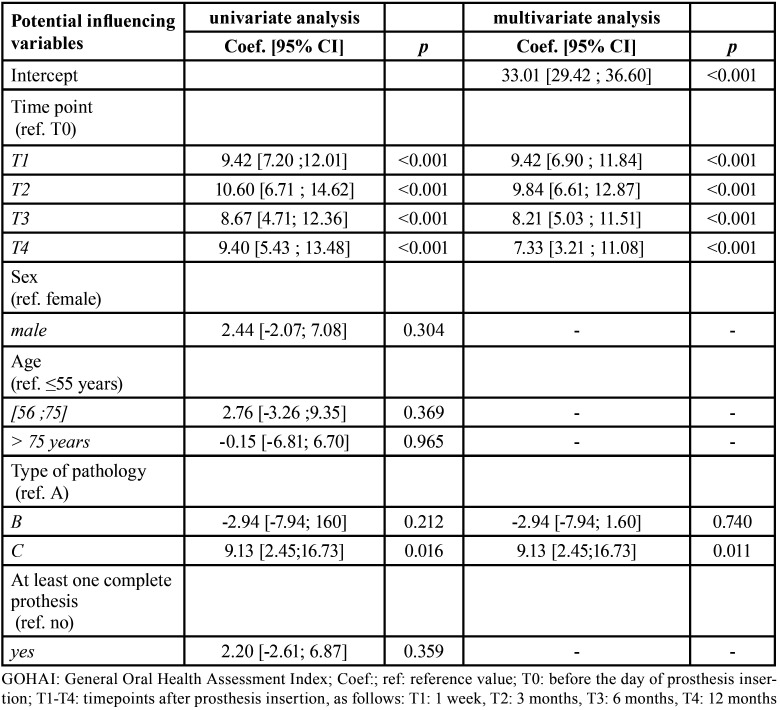
Mixed linear regression analysis results show associations between the overall GOHAI score and potential influencing factors.

**Table 4:**
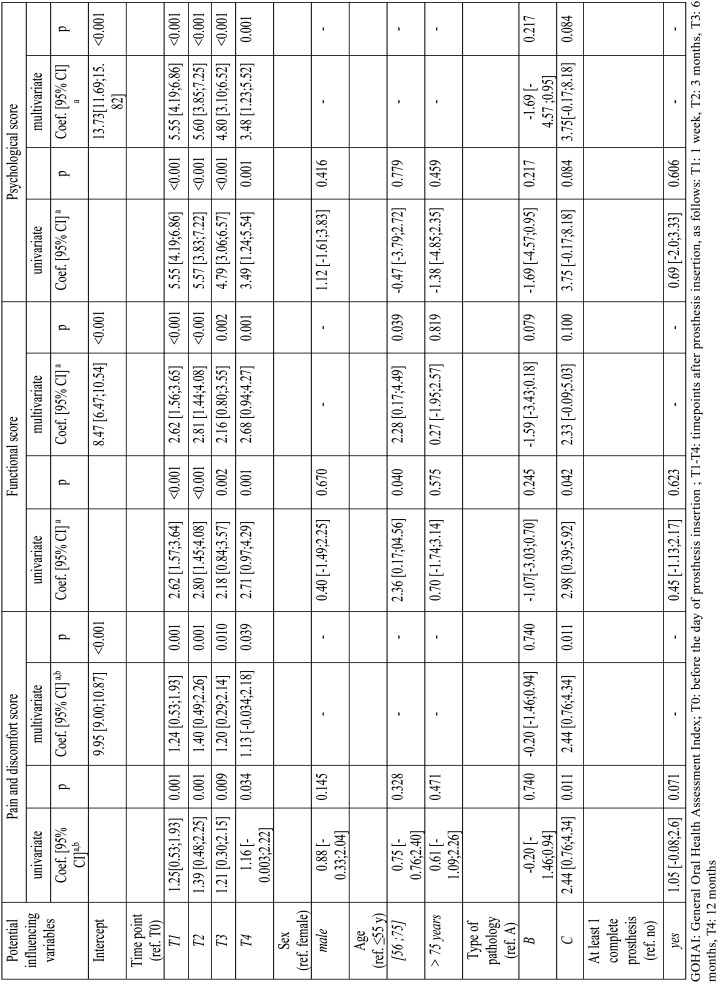
Mixed linear regression analysis results show associations between each GOHAI domain-score and potential influencing factors.

In the multivariate analysis, we compared the overall GOHAI score and each domain-score between baseline (T0) and each post-insertion (post-operative) time-point (T1, T2, and T3). We observed significant improvements in all scores over time. Compared to the scores at T0, we observed increases at T1 in the GOHAI-add score, the pain and discomfort score, the functional score, and the psychosocial score, by score increases of 9.42 6.90;11.84., 1.24 0.53;1.93., 2.62 1.56;3.65., and 5.55 4.19;6.86., respectively.

The GOHAI-add score was significantly higher among patients in group C than in patients in group A, by a score increase of 9.130 2.45;16.73. (Table 3). This effect was largely due to the significant change in the pain and discomfort score in group C, which changed by a score increase of 2.44 0.76;4.34. compared to patients in group A (Table 4).

Interestingly, compared to patients under 55 years old, patients between 55 and 75 years old had significantly higher functional scores (coefficient = 2.28 0.17;4.49.). Moreover, there was no significant interaction with time during the 1-year follow-up.

## Discussion

This study showed that prosthetic dental rehabilitation improved the functional and aesthetic qualities of life in patients with HNC that underwent irradiation. This study compared quality of life before and after prosthesis placement, as recommended by some authors (11). The original nature of this work was the focus on the evolution of quality of life, and the effects of specific factors, including tumour location and the type of removable dental prosthesis.

Although the GOHAI questionnaire was not specifically designed for HNC, we chose it to assess improvements in oral quality of life (16,17), because it was the only French language questionnaire specific to dental pathologies, which explicitly addressed oral rehabilitation (18). We chose a 1-year observation period, with measurements at different time-points, because it was well suited to our purpose. First, quality of life has been shown to decrease within 3 months after the end of treatment, and second, quality of life can return to a baseline level within 12 months after the completion of a therapeutic protocol. On the other hand, no significant difference in the level of quality of life has been reported between 12 and 36 months after a treatment protocol (19). Therefore, we were interested in this 1-year post-treatment (surgery, radio-chemotherapy) period to evaluate the effect of rehabilitation on the patient’s quality of life as early as possible after treatment. We found that the GOHAI-add scores increased significantly between T0 and T1 and between T0 and T2. However, after that, the scores were stable, although they decreased slightly at T3 and T4, compared to T1 and T2. Therefore, the quality of life showed an immediate improvement after the insertion of the rehabilitation prosthesis. This improvement remained sTable over time, from T2 onwards and did not improve further.

Like the GOHAI-add score, the three domain-scores (psychosocial, functional, and discomfort-pain) increased after T0, but at different time-points. The psychosocial component score increased between T0 and the other time-points. This score increased more significantly than the functional and discomfort-pain scores. The domain-scores remained stable between T1 and T4, with less important changes between T3 and T4.

All these results were consistent with those obtained in a preliminary study; both studies demonstrated the importance of assessing the different domains related to quality of life (13). The hypothesis that improvements in quality of life were mainly linked to the psychosocial, social relationships, and self-esteem domains appeared to be confirmed over time. For example, Patterson *et al*. showed that loneliness was linked to the depressive state in HNC survivors (20). They showed that quality of life improved when self-esteem and social relationships improved. In the present study, we found that improvements in the GOHAI-add score remained stable over time. Moreover, the different domains varied in the same direction, which excluded that compensatory changes in the different domains might explain the stability in the overall score.

The main limitation of the current study was the medium-to-small sample size, particularly at T4. This feature might have weakened the statistical power of the study. However, the use of a linear mixed model analysis allowed us to exploit the all the data collected, and it provided reliable results. In addition, we minimized potential practitioner-dependent bias, because the same practitioner performed all questionnaire administrations, all the data collection, and performed all prosthetic rehabilitations with the same techniques.

Previously, Ciocca showed that the quality of life was not different between patients with HNC that received fixed implant-supported and removable prosthetic rehabilitations after mandibular surgery with flap reconstructions (21). However, removable prostheses were the preferred option for patients with a high risk of developing osteoradionecrosis (10).

According to Petrosyan *et al*., there is no proven link between prosthetic rehabilitation and improved quality of life; however, quality of life may improve independently over time (22). Our findings showed that quality of life improved within a short time after a prosthesis insertion; moreover, our results excluded an influence of time on this improvement. This finding was consistent with that noted by Dholam *et al*., who studied patients that wore a prosthesis for 12 months (12). Additionally, our study clarified the conditions of improvement; we found that, among patients that received a removable prosthesis, there was no significant difference between those that wore at least one full denture and those that wore partial dentures. All the patients in our sample had undergone radiotherapy. Therefore, the corresponding side effects (xerostomia, burns, etc.) were common to both groups. This commonality may explain the lack of significant difference between the partial and total edentulous groups (23). Future studies might confirm this hypothesis, by comparing improvements between irradiated and non-irradiated groups, based on strictly qualitative surveys to gain better feedback regarding prosthesis rehabilitation (11).

The use of the ICD-11 to classify the primary tumour location was relevant, because patients could be grouped according to the anatomic consequences of the therapeutic protocol. Similar disabilities have an equivalent functional and social impact (11). In this study, we found that patients with a primary tumour in a laryngeal or pharyngeal location (Group C) had better GOHAI-add scores than those with a primary tumour in a maxillary location (Group A). In contrast, patients with a primary tumour localised to the mandible (Group B) had worse GOHAI-add scores than those with a primary tumour in a maxillary location. These results might be explained by the fact that disFigurement and stigmata after the therapeutic protocol have the greatest aesthetic impact when they are localised to the maxilla or mandible. In particular, patients have reported that surgical procedures, like a mandibulectomy, glossectomy, or extensive maxillectomy, had marked negative influences on quality of life (3) (24). In our study, these aesthetic problems were less marked for patients with pathologies in laryngeal or pharyngeal locations. This finding may explain, at least in part, the better scores for the different questionnaire components among patients treated for laryngeal or pharyngeal pathologies, compared to those treated for maxillary pathologies.

In conclusion, this study showed that prosthetic rehabilitation had a sustainable influence on the quality-of-life evolution. In particular, prosthetic rehabilitation influenced the psychosocial component (social relationships, self-esteem) in the first weeks after the insertion of the prosthesis, which notably improved quality of life. The type of removable prosthesis did not seem to influence the results obtained. However, primary tumours in a mandibular location had a negative influence on quality of life. These results should be confirmed in future studies with larger samples that allow the exploitation of all the data collected.

## References

[1] Cowppli-Bony A, Colonna M, Ligier K, Jooste V, Defossez G, Monnereau A (2019). Descriptive epidemiology of cancer in metropolitan France: Incidence, survival and prevalence. Bull Cancer.

[2] Quadri MF, Alamir AW, John T, Nayeem M, Jessani A, Tadakamadla SK (2020). Effect of prosthetic rehabilitation on oral health-related quality of life of patients with head and neck cancer: a systematic review. Transl Cancer Res.

[3] Zhang Y, Cui C, Wang Y, Wang L (2020). Effects of stigma, hope and social support on quality of life among Chinese patients diagnosed with oral cancer: a cross-sectional study. Health Qual Life Outcomes.

[4] Klein J, Livergant J, Ringash J (2014). Health related quality of life in head and neck cancer treated with radiation therapy with or without chemotherapy: a systematic review. Oral Oncol.

[5] Moore KA, Ford PJ, Farah CS (2014). Support needs and quality of life in oral cancer: a systematic review. Int J Dent Hyg.

[6] Le Corroller-Soriano AG, Bouhnik AD, Preau M, Malavolti L, Julian-Reynier C, Auquier P (2011). Does cancer survivors' health-related quality of life depend on cancer type? Findings from a large French national sample 2 years after cancer diagnosis. Eur J Cancer Care (Engl).

[7] Sayed SI, Elmiyeh B, Rhys-Evans P, Syrigos KN, Nutting CM, Harrington KJ (2009). Quality of life and outcomes research in head and neck cancer: a review of the state of the discipline and likely future directions. Cancer Treat Rev.

[8] Petersen PE (2003). The World Oral Health Report 2003: continuous improvement of oral health in the 21st century--the approach of the WHO Global Oral Health Programme. Community Dent Oral Epidemiol.

[9] Vosselman N, Alberga J, Witjes MHJ, Raghoebar GM, Reintsema H, Vissink A (2021). Prosthodontic rehabilitation of head and neck cancer patients-Challenges and new developments. Oral Dis.

[10] Wiedenmann F, Liebermann A, Probst F, Troeltzsch M, Balermpas P, Guckenberger M (2020). A pattern of care analysis: Prosthetic rehabilitation of head and neck cancer patients after radiotherapy. Clin Implant Dent Relat Res.

[11] Abed H, Burke M, Fenlon MR, Scambler S, Scott SE (2020). Use of dentures, receipt of information, quality of life and oral function following radiotherapy for head and neck cancer. Spec Care Dentist.

[12] Dholam K, Chouksey G, Dugad J (2020). Impact of Oral Rehabilitation on Patients with Head and Neck Cancer: Study of 100 Patients with Liverpool Oral Rehabilitation Questionnaire and the Oral Health Impact Profile. Indian J Otolaryngol Head Neck Surg.

[13] Silvestri F, Saliba-Serre B, Graillon N, Fakhry N, Ruquet M, Maille G (2021). Quality of life in irradiated patients with head and neck cancer: A preliminary study about the impact of prosthetic rehabilitation. J Clin Exp Dent.

[14] World Medical Association (2013). World Medical Association Declaration of Helsinki: ethical principles for medical research involving human subjects. JAMA.

[15] (2019/2021). International Classification of Diseases, Eleventh Revision (ICD-11). World Health Organization.

[16] Atchison K, Dolan TA (1990). Development of the geriatric oral health assessment index. J Dent Educ.

[17] Tubert-Jeannin S, Riordan PJ, Morel-Papernot A, Porcheray S, Saby-Collet S (2003). Validation of an oral health quality of life index (GOHAI) in France. Community Dent Oral Epidemiol.

[18] Heutte N, Plisson L, Lange M, Prevost V, Babin E (2014). Quality of life tools in head and neck oncology. Eur Ann Otorhinolaryngol Head Neck Dis.

[19] Murphy BA, Ridner S, Wells N, Dietrich M (2007). Quality of life research in head and neck cancer: a review of the current state of the science. Crit Rev Oncol Hematol.

[20] Patterson JM, Lu L, Watson LJ, Harding S, Ness AR, Thomas S (2021). Associations between markers of social functioning and depression and quality of life in survivors of head and neck cancer: findings from the Head and Neck Cancer 5000 study. Psychooncology.

[21] Ciocca L, Tarsitano A, Mazzoni S, Gatto MR, Marchetti C, Scotti R (2015). Evaluation of Masticatory Efficiency and QoL Improvements After Prosthetic Rehabilitation of Mandibular Cancer Patients Reconstructed with a Fibula Free Flap. Int J Prosthodont.

[22] Petrosyan V, Ball D, Harrison R, Ameerally P (2016). Among Patients Undergoing Ablative Treatment for Oral Cancer, Does the Provision of Oral Rehabilitation Improve the Quality of Life? A Review of the Current Literature. J Oral Maxillofac Surg.

[23] Fromm L, Gotfredsen K, Wessel I, Øzhayat EB (2019). Oral health-related quality of life, oral aesthetics and oral function in head and neck cancer patients after oral rehabilitation. J Oral Rehabil.

[24] Morimata J, Otomaru T, Murase M, Haraguchi M, Sumita Y, and Taniguchi H (2013). 2013. Investigation of factor affecting health-related quality of life in head and neck cancer patients. Gerodontology.

